# Platelet Count to Spleen Diameter Ratio for the Diagnosis of Gastroesophageal Varices in Liver Cirrhosis: A Systematic Review and Meta-Analysis

**DOI:** 10.1155/2017/7407506

**Published:** 2017-02-08

**Authors:** Runhua Chen, Han Deng, Xia Ding, Chune Xie, Wei Wang, Qian Shen

**Affiliations:** ^1^Department of Gastroenterology, Dongfang Hospital, Beijing University of Chinese Medicine, Beijing, China; ^2^Postgraduate College, Dalian Medical University, Dalian, China; ^3^Department of Hospital Administration, Beijing University of Chinese Medicine, Beijing, China; ^4^Department of Personnel, Dongfang Hospital, Beijing University of Chinese Medicine, Beijing, China; ^5^Department of Cardiology, Wangjing Hospital, China Academy of Chinese Medical Sciences, Beijing, China; ^6^Department of Massage and Physiotherapy, Dongfang Hospital, Beijing University of Chinese Medicine, Beijing, China

## Abstract

Platelet count to spleen diameter ratio (PSR) was studied extensively as a noninvasive method of diagnosis for varices. The present study aimed to systematically assess the performance of PSR in the diagnosis of varices. PubMed, EMBASE, and article references were searched. The summary receiver operating characteristic curves (AUSROCs), sensitivities, specificities, positive and negative likelihood ratio, and diagnostic odds ratio were calculated. The heterogeneity, quality, and publication bias of studies were evaluated. Subgroup and sensitivity analyses were performed. A total of 49 papers were included. The AUSROCs of PSR for any varices and high-risk varices were 0.8719 and 0.8132, respectively. The summary sensitivities of PSR for any varices and high-risk varices were 0.84 and 0.78, respectively. The summary specificities of PSR for any varices and high-risk varices were 0.78 and 0.67, respectively. The AUSROC of PSR for any varices at the threshold of 909 was 0.8867. The AUSROC of PSR for any varices in viral liver cirrhosis was 0.8675. The overall quality of studies was moderate. Significant heterogeneity and publication bias existed in the study. In conclusion, PSR can be used to identify varices in liver cirrhosis. PSR had a high sensitivity in viral liver cirrhosis.

## 1. Introduction

Gastroesophageal varices are one of the major complications of liver cirrhosis. Early detection of varices in cirrhotic patients is crucial to ensure timely initiation of prophylactic therapies. Platelet count to spleen diameter ratio (PSR) was first proposed by Giannini et al. to predict the presence of varices in 2003 [1]. An increasing number of studies have since evaluated the accuracy of PSR in the detection of varices, albeit drawing inconsistent conclusions. In two previous meta-analyses regarding the diagnostic accuracy of PSR in predicting the presence of varices, Ying et al. [2] recommended using PSR to identify varices to decrease the use of upper gastrointestinal endoscopy, while Chawla et al. [3] found that PSR has low grade evidence to replace upper gastrointestinal endoscopy as a noninvasive method for varices. The performance of PSR for varices is still not unified at present, which has limited the use of PSR in clinical practice. Thus, we conducted this systematic review and meta-analysis to evaluate the performance of PSR for varices.

## 2. Methods

Selection, data extraction, and quality assessment of studies were conducted by two investigators (RC and QS) independently. Disagreement between the two investigators was resolved by a consensus.

### 2.1. Search Strategy and Selection Criteria

PubMed and EMBASE were searched on May 27, 2016. The search terms were as follows: (((((((platelet count to spleen diameter ratio) OR PSR) OR PC/SD)) OR ((((platelet) OR platelet count)) AND ((spleen) OR spleen diameter)))) AND liver cirrhosis) AND varices. Relevant references were also screened. Duplicates, commentaries, reviews, case reports, letters, meta-analyses, book sections, and meeting abstracts were excluded. The inclusion criteria were as follows: (1) participants should be diagnosed with liver cirrhosis; (2) upper gastrointestinal endoscopy should be performed as the reference tests for the diagnosis of varices; (3) PSR should be performed as alternative tests for the diagnosis of varices; (4) diagnostic accuracy data of PSR on the diagnosis of varices were available. The language and publication year were not limited.

### 2.2. Data Extraction

The following data from each study was extracted: the first author, publication year, region, study design, total number of patients, age, sex, etiology of liver cirrhosis, hepatocellular carcinoma (HCC), Child-Pugh class, location of varices (i.e., esophageal varices [EV] and/or gastric varices [GV]), prevalence of any varices and/or high-risk (or large) varices, cut-off value, true positive (TP) value, false positive (FP) value, false negative (FN) value, and true negative (TN) value. Missing values were calculated using the following formulae: sensitivity = TP/(TP + FN), and specificity = TN/(TN + FP). The raw TP, FN, FP, and TN numbers of included studies were shown in Supplementary Table 1 (see Supplementary Material available online at https://doi.org/10.1155/2017/7407506).

### 2.3. Quality Assessment

The quality of each study was assessed by QUADAS-2 (Quality Assessment of Diagnostic Accuracy Studies-2) tool [4]. This tool comprises four domains: patient selection, index test, reference standard, and flow and timing. The risk of bias in each domain was rated as “low risk,” “high risk,” and “unclear risk” with signaling questions. The applicability concerns in the first three domains were assessed as “low concern,” “high concern,” and “unclear concern.” If the total number of “low risk” and “high concern” was equal or greater than 6 in a study, the study was considered as high quality.

### 2.4. Statistical Analysis

The area under the summary receiver operating characteristic curves (AUSROCs) with standard errors (SEs) and *Q* indexes with SEs, summary sensitivities and specificities with 95% confidence intervals (CIs), summary positive and negative likelihood ratios (PLRs and NLRs) with 95% CIs, and summary diagnostic odds ratios (DORs) with 95% CIs were calculated using statistical software (Meta-Disc software version 1.4). We analyzed these data using the random-effects model. The diagnostic threshold was analyzed by Spearman correlation coefficient and *p* value. *p* < 0.05 showed a statistically significant diagnostic threshold effect. Therefore, only AUSROCs with SEs and *Q* indexes with SEs were calculated. The heterogeneity among studies was evaluated by Chi-square test and inconsistency index. A statistically significant heterogeneity was defined as *p* < 0.1 and/or *I*^2^ > 50%.

To explore the publication bias, we performed Deeks' funnel plot asymmetry test in Stata 12.0 (College Station, TX, USA). Sensitivity analyses were performed via removing each study to evaluate the impact on the pooled results of the removed study.

We calculated the diagnostic accuracy of PSR for various cut-off values in predicting the presence of any and high-risk varices. If there were multiple different cut-off values in the same paper, we selected the optimal cut-off values. PSR for any varices at threshold of 909 was the most generally accepted cut-off value at present. Thus, we performed subgroup analyses using the cut-off value of 909. In addition, we performed subgroup analyses based on the etiology of cirrhosis, region, study design, prevalence of varices, sample size, and study quality for any varices.

## 3. Results

### 3.1. Selection of Studies

**Figure 1:**
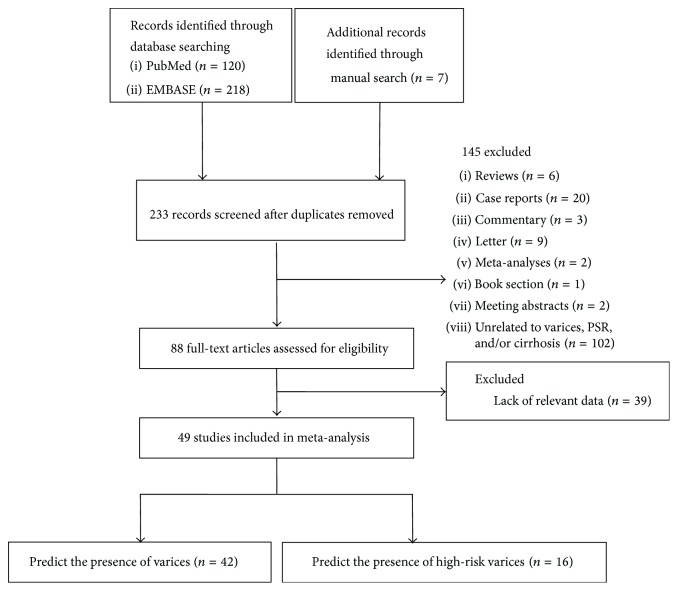
Flowchart of study selection.

A total of 345 papers were selected from PubMed (*n* = 120), EMBASE (*n* = 218), and manual search (*n* = 7). The flow diagram of study selection was shown in Figure 1. 112 duplicates and 145 irrelevant papers were excluded. Then, we screened 88 full-text articles for eligibility. 39 papers which lacked relevant diagnostic data were excluded. Among them, 28 papers were applied only with abstracts. Finally, 49 papers [1, 5–52] were included in our study. 42 and 16 papers were about any varices and high-risk varices, respectively.

### 3.2. Characteristics of Studies

The characteristics of studies were shown in Table 1. Our meta-analysis included a total of 6274 patients. 22 papers were prospective studies. 2 papers [10, 20] were published in abstracts and 47 papers were full-texts. The etiologies of cirrhosis were alcohol, schistosomiasis, and viral hepatitis in 1 [12], 2 [5, 32], and 13 [9, 19, 22, 26, 27, 30, 33, 36, 39, 40, 42–44] papers, respectively. Two papers were about EV and GV [8, 12], and the rest of the papers were about EV alone. In two papers, all patients had Child-Pugh A [22, 40]. The diagnostic accuracy of PSR for any varices at threshold of 909 was reported in 19 papers.

### 3.3. Quality Assessment of Studies

The overall quality of the included studies was not very high (Supplementary Table 2). In the patient selection domain, only 17 papers were rated as “low risk.” In the index test and reference standard domains, 10 and 14 papers were rated as “low risk.” Most papers had not reported whether investigators were blinded when interpreting the results of index test and reference standard. 2 papers were rated as “high risk” in flow and timing domain as the interval time between index test and reference standard was greater than 3 months. 44 papers have “high concern” in patient selection domain. In addition, all papers were rated as “high concern” in index test and reference standard domains. 11 papers were considered with high quality.

### 3.4. Overall Results

Significant threshold effect was not found in overall meta-analyses.

**Figure 2:**
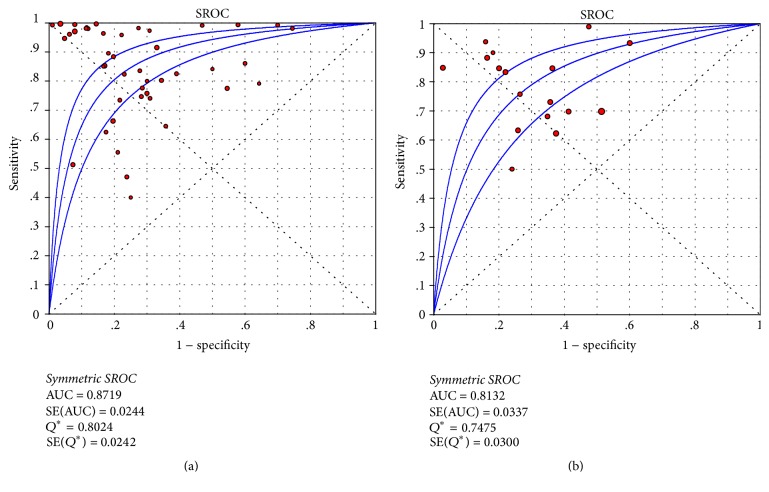
AUSROCs of PSR for varices in liver cirrhosis. (a) Any size varices; (b) high-risk varices.

**Figure 3:**
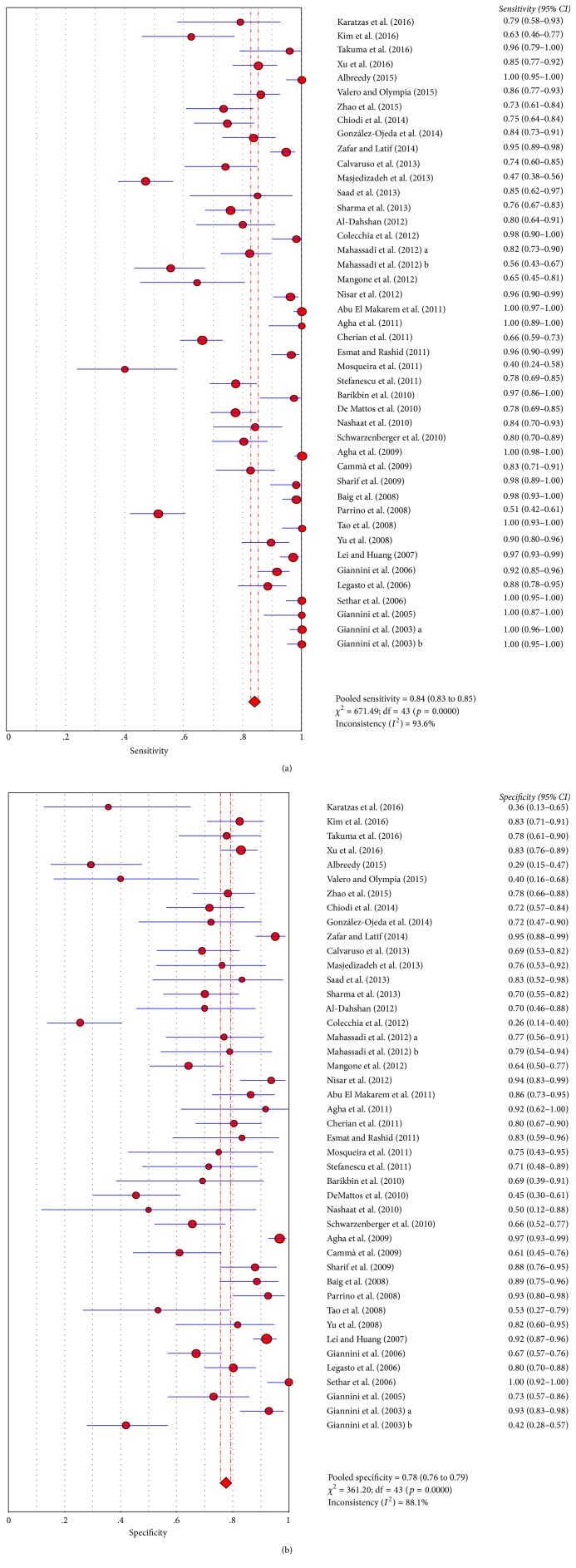
Summary sensitivity and specificity of PSR for any size varices in liver cirrhosis. (a) Summary sensitivity; (b) summary specificity.

Diagnostic accuracy of PSR for the presence of any varices was pooled from 42 papers [1, 5–10, 13, 14, 16–19, 21–34, 36–40, 42–50, 52]. The AUSROC was 0.8719 (Figure 2(a)). The summary sensitivity and specificity were 0.84 (95% CI: 0.83–0.85) and 0.78 (95% CI: 0.76–0.79), respectively (Figure 3). The summary PLR, NLR, and DOR were 3.54 (95% CI: 2.75–4.56), 0.17 (95% CI: 0.12–0.23), and 25.32 (95% CI: 15.72–40.77), respectively.

**Figure 4:**
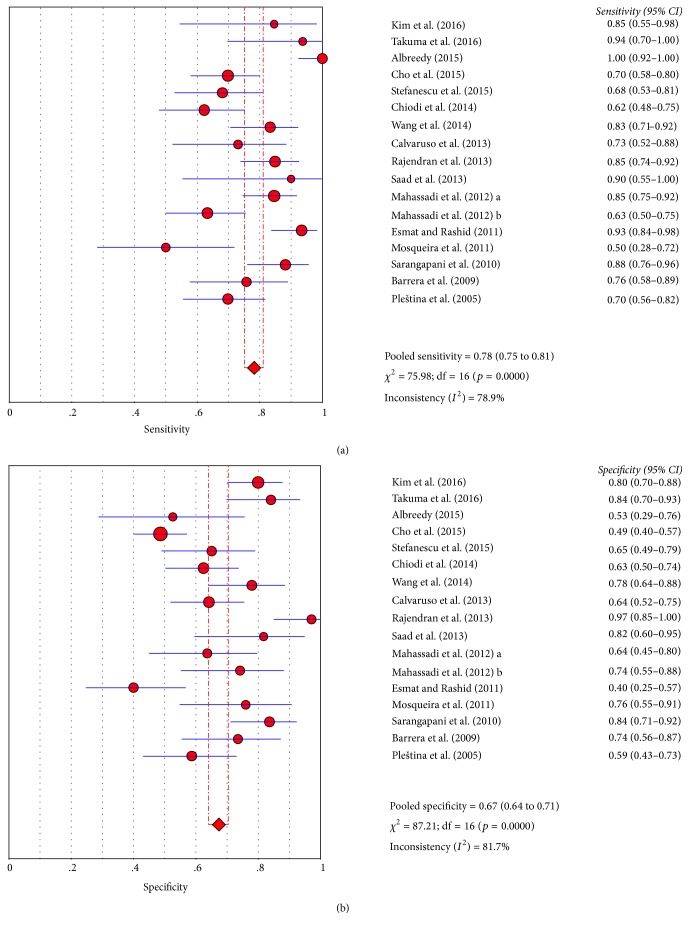
Summary sensitivity and specificity of PSR for high-risk varices in liver cirrhosis. (a) Summary sensitivity; (b) summary specificity.

Diagnostic accuracy of PSR for the presence of high-risk varices was pooled from 16 papers [6, 7, 11–13, 15, 17, 19, 20, 22, 25, 29, 30, 35, 41, 51]. The AUSROC was 0.8132 (Figure 2(b)). The summary sensitivity and specificity were 0.78 (95% CI: 0.75–0.81) and 0.67 (95% CI: 0.64–0.71), respectively (Figure 4). The summary PLR, NLR, and DOR were 2.54 (95% CI: 1.99–3.24), 0.32 (95% CI: 0.24–0.44), and 9.08 (95% CI: 5.33–15.47), respectively. The diagnostic accuracy of PSR for high-risk varices was lower than PSR for any varices.

### 3.5. Subgroup Results

The subgroup results were summarized in Table 2. Significant threshold effect was found in the subgroup of South America. Thus, their diagnostic accuracy was not combined.

### 3.6. Heterogeneity

Significant heterogeneity between papers was found in most analyses except for the subgroup of North America and sample size less than 100.

### 3.7. Sensitivity Analyses

Sensitivity analysis results were similar to the overall meta-analysis results. The heterogeneity remained significant (data not shown).

### 3.8. Publication Bias

The publication bias existed in the study (*p* = 0.007).

## 4. Discussions

In our study, the AUSROC of PSR for any varices was 0.8719. The summary sensitivity and specificity were 0.84 and 0.78, respectively. The diagnostic accuracy of PSR for high-risk varices was lower than PSR for any varices. The diagnostic accuracy of PSR for varices at threshold of 909 was similar to PSR at various thresholds. While the summary sensitivity (0.92) in viral liver cirrhosis was improved over that of mixed etiologies, they had the same summary specificities (0.78). The subgroup analysis of Asia had the highest AUSROC (0.9195).

The high diagnostic accuracy of PSR for varices can be explained as follows. Varices and hypersplenism are the results of portal hypertension. The platelet count can be influenced by many factors in cirrhotic patients other than hypersplenism. The decreased thrombopoietin production is the reason. Thrombopoietin is mainly produced by hepatocytes and the quantity can be largely reduced when the hepatocytes was damaged. In addition, the shortened platelet mean lifetime and myelotoxic effects of alcohol or hepatitis viruses also reduced the platelet count. Splenomegaly is the clinical manifestation of hypersplenism. Thus, a combined index of platelet count and spleen diameter has much more relevance with portal hypertension and varices than the sole decreased platelet count [1].

In clinic practice, the measure of spleen diameter and platelet count is easily obtainable during the routine ultrasonography and serum examination. PSR is convenient, cheap, and noninvasive. Based on our study, we recommend that those patients whose PSR is less than 909 should undergo upper gastrointestinal endoscopy to evaluate the grade of varices. 80% of patients whose value of PSR is greater than 909 can avoid unnecessary upper gastrointestinal endoscopic examination.

Compared with the two previous studies, our meta-analysis included a greater number of studies without limiting the publication language and cut-off values. Compared with other noninvasive methods, PSR has an upper-middle performance for varies. As previous studies have shown [53–55], serum markers cannot be used to identify varices for the low-moderate diagnostic accuracy. Computer tomography has similar summary sensitivity (0.896) and specificity (0.723) compared to PSR [56]. In addition, the diagnostic accuracy of PSR for varices was slightly higher than spleen stiffness measurement [57] and liver stiffness measurement [58]. Their summary sensitivities were 0.78 and 0.87, respectively. The summary specificities were 0.76 and 0.53, respectively. While the diagnostic accuracy of PSR for varices was slightly lower than capsule endoscopy [59], its summary sensitivity and specificity were 0.85 and 0.84, respectively. Some studies reported that splenoportal index and congestion index have high diagnostic accuracy. Their sensitivities and specificities were both greater than 80% [60, 61]. However, there are no systematic studies to evaluate their performance of varices.

Our study has some limitations. (1) 39 papers lacking relevant data were excluded, out of which some reported that PSR had no statistically significant difference in predicting the presence of varices. (2) Most analyses had significant heterogeneity. It may be attributed to the different selection criteria of patients, such as the prevalence of decompensated cirrhosis, etiologies of cirrhosis, and history of variceal bleeding. We have no evidence to support this assumption. The same situation was also found in the previous meta-analyses [2, 3]. (3) The publication bias exists in the study. (4) Most TP, FP, FN, and TN were recalculated using sensitivities and specificities, which may introduce some errors. (5) PSR is not applicable to patients with a history of splenectomy.

In conclusion, PSR can be used to identify varices in liver cirrhosis. PSR had a high sensitivity in viral liver cirrhosis.

## Supplementary Material

Supplementary Material includes two parts. Supplementary Table 1 described the diagnostic value of included studies. Supplementary Table 2 described the QUADAS-2 results.

## Figures and Tables

**Table 1:** Characteristics of studies.

First author (year)	Regions	Study design	Number of total Pts	Age (year)	Male (%)	Etiology of cirrhosis	HCC (%)	Child-Pugh class (%)	Location of varices	Prevalence of varices (%)	Prevalence of high-risk/large varices (%)	Cut-off of varices
Karatzas (2016)	Greece	Prospective	38	63	78.9%	Alcohol 47.4%	NA	A, 55.3%	EV + GV	63.2%	10.5%	1310.597 (optimal)
						Viral hepatitis 34.2%		B, 28.9%				
						Others 18.4%		C, 15.8%				909

Kim (2016)	South Korea	Retrospective	103	53.5 ± 11.8	65.0%	Alcohol 28.2%	NA	NA	EV	38.8%	12.6%	860
						HBV 50.5%						
						HCV 8.7%						
						HBV and alcohol 7.8%						
						Others 4.9%						

Takuma (2016)	Japan	Prospective	60	70.8 ± 9.9	56.7%	Alcohol 10%	NA	A, 68.3% B, 30% C, 1.7%	EV	40.0%	26.7%	3.36
						HBV 13.3%						
						HCV 58.3%						
						Others 18.3%						

Xu (2016)	China	Prospective	236	61.4 ± 10.2	43.2%	Schistosomiasis 100%	NA	NA	EV	40.3%	NA	1004 (optimal)
												909

Albreedy (2015)	Egypt	Prospective	100	44.24 ± 7.05	58.0%	NA	0%	A, 41%	EV	66.0%	47.0%	979.9
								B, 33%				
								C, 26%				

Cho (2015)	South Korea	Retrospective	219	52/50^*∗*^	91.8%	Alcohol 100%	0%	A, 59.4%	EV + GV	NA	33.3%	NA
								B, 36.1%				
								C, 4.6%				

Stefanescu (2015)	Romania	Cross-sectional	90	56.47 ± 9.38/54.98 ± 8.42^*∗*^	55.6%	Alcohol 33.3%	0%	A, 62.2% B, 35.6% C, 2.2%	EV	81.1%	52.2%	NA
						HBV 13.3%						
						HCV 33.3%						
						Others 20%						

Valero (2015) (abstract)	Philippines	Retrospective	101	64.8/61.6	NA	NA	0%	NA	EV	85.1%	NA	1.86

Zhao (2015)	China	Retrospective	124	51.34 ± 11.089	57.3%	HCV 100%	NA	NA	EV	51.6%	NA	909

Chiodi (2014)	Uruguay	Retrospective	125	54	56.8%	Alcohol 40%	0%	NA	EV	63.2%	42.4%	1010%
						HBV 3.2%						
						HCV 16.8%						
						Autoimmune 12%						
						Others 19.2%						
						Unknown 8.8						

González-Ojeda (2014)	Mexico	Cross-sectional	91	53.75 ± 12	54.9%	Alcohol 52.7%	0%	A, 18.7% B, 40.7% C, 40.7%	EV	80.2%	57.1%	884
						HCV 26.4%						
						Others 11%						
						Unknown 10%						

Wang (2014)	China	Retrospective	104	59	56.7%	Alcohol 17.3%	NA	A, 24% B, 35.6% C, 40.4%	EV	99.0%	51.9%	NA
						HBV 66.3%						
						Alcohol and HBV 12.5%						
						Unknown 3.8%						

Zafar (2014)	Pakistan	Prospective	215	46.93 ± 13.22	42.3%	NA	NA	NA	EV	60.9%	NA	909

Calvaruso (2013)	Italy	Prospective	96	63.2 ± 9.5	69.8%	HCV 100%	0%	A, 100%	EV	56.3%	27.1%	800

Masjedizadeh (2013)	Iran	Prospective	140	57/53	70.0%	Alcohol 3.6%	NA	A, 43.6% B, 42.1% C, 14.3%	EV	85.0%	33.6%	663
						HBV 36.4%						
						HCV 17.1%						
						Autoimmune 8.6%						
						Others 5.7%						
						Unknown 28.6%						

Rajendran (2013) (abstract)	India	Cross-sectional	101	NA	93.1%	Alcohol 85%	NA	NA	EV	95.0%	65.0%	NA
						HBV 9%						
						HCV 5%						

Saad (2013)	Egypt	NA	32	55 ± 6.6/49.5 ± 4.7/	62.5%	HCV 100%	0%	A, 71.9%	EV	62.5%	31.3%	545
				48.9 ± 4.7^*∗*^				B, 28.1%				

Sharma (2013)	India	NA	174	49.3 ± 11.7	88.5%	Alcohol 44.3%	0%	A, 31.6% B, 56.9% C, 11.5%	EV	71.3%	44.8%	1023.2 (optimal) 909
						HBV 13.2%						
						HCV 16.7%						
						Unknown 25.9%						

Al-Dahshan (2012)	Egypt	NA	60	52.62 ± 8.22	78.3%	HBV 81.7%	0%	NA	EV	66.7%	NA	1023
						HCV 18.3%						

Colecchia (2012)	Italy	Prospective	100	54	71.0%	HCV 100%	0%	A, 68% B, 32%	EV	53.0%	49.0%	1883 (optimal)
												513

Mahassadi (2012)	Cote d'Ivoire	NA (training sample)	111	49	70.3%	Alcohol 20.7% HBV 61.3% HCV 12.6% Others 5.4%	0%	A, 22.5% B, 35.1% C, 42.3%	EV	76.6%	70.3%	868
		NA (validation sample)	91	50	64.2%	Alcohol 26.4% HBV 58.2% HCV 15.4%	0%	A, 19.8% B, 52.7% C, 22% Unknown, 5.5%	EV	79.1%	65.9%	868

Mangone (2012)	Italy	Prospective	87	62.8	58.6%	Alcohol 8%	NA	A, 90.8% B, 6.9% Unknown, 2.3%	EV	35.6%	NA	936.364 (optimal) 909
						HBV 10.5%						
						HCV 63.2%						
						Others 18.3%						

Nisar (2012)	Pakistan	Cross-sectional	150	50.99 ± 12.99	54.0%	NA	NA	NA	EV	68.0%	NA	909

Abu El Makarem (2011)	Egypt	Prospective	175	48	65.7%	HCV 100%	NA	A, 26.3%	EV	74.9%	NA	939.7
								B, 33.7%				
								C, 40%				

Agha (2011)	Italy	Prospective	43	61	70.0%	Schistosomiasis 100%	NA	NA	EV	72.1%	44.2%	885

Cherian (2011)	India	Prospective	229	42	61.6%	Alcohol 42.4%	0%	A, 18.3% B, 55.5% C, 26.2%	EV	77.7%	35.4%	666
						HBV 15.3%						
						HCV 10%						
						Others 12.7%						
						Unknown 19.7%						

Esmat (2011)	Egypt	Prospective	100	49.2 ± 8	48.0%	HCV 100%	NA	A, 20%	EV	82.0%	60.0%	1326.6 (optimal) 909
								B, 31%				
								C, 49%				

Mosqueira (2011)	Peru	Retrospective	47	60.74	50.0%	Alcohol 25.5%	NA	NA	EV	74.5%	46.8%	909
						HBV 2.1%						
						HCV 14.9%						
						Autoimmune 8.5%						
						Unknown 48.9%						

Stefanescu (2011)	Romania	Prospective	137	56	56.2%	Alcohol/HCV 100%	NA	A, 64.9%	EV	84.9%	44.0%	1068
								B, 28.4%				
								C, 6.8%				

Barikbin (2010)	Iran	Prospective	50	52.1 ± 16.2	82.0%	Alcohol 4%	0%	A, 10% B, 28% C, 62%	EV	74.0%	62.0%	921
						HBV 38%						
						HCV 14%						
						Others 6%						
						Unknown 38%						

De Mattos (2010)	Brazil	NA	164	56.6 ± 11.6	56.7%	Alcohol 29.3%	NA	A, 57.6% B, 37.7% C, 4.6%	EV	73.2%	40.8%	909
						Viral hepatitis 43.9%						
						Viral hepatitis and alcohol 10.4%						
						Others 16.5%						

Nashaat (2010)	Egypt	NA	50	49.6 ± 8.8	74.0%	HBV 20%	NA	A, 38%	EV	88.0%	NA	820
						HCV 70%		B, 42%				
						HBV and HCV 10%		C, 20%				

Sarangapani (2010)	India	Prospective	106	45	67.9%	Alcohol 58.5%	0%	NA	EV	72.6%	48.1%	NA%
						HBV 21.7%						
						Others 19.8%						

Schwarzenberger (2010)	US	Retrospective	137	56	64.0%	Alcohol 18%	NA	NA	EV	55.5%	18.2%	909
						HBV 23%						
						HCV 34%						
						Others 17%						
						Unknown 9						

Agha (2009)	Pakistan	Prospective	311	49	55.6%	HCV 100%	0%	A, 25.8%	EV	49.5%	12.9%	909
								B, 58.6%				
								C, 15.6%				

Barrera (2009)	Chile	Prospective	67	66 ± 12.2	43.3%	Alcohol 26.9%	NA	A, 46.2% B, 38.8% C, 15%	EV	85.1%	49.3%	NA
						Viral hepatitis 7.5%						
						PBC 14.9%						
						Others 26.8%						
						Unknown 26.9%						

Cammà (2009)	Italy	Prospective	104	61.4 ± 9.5	57.7%	HCV 100%	0%	A, 100%	EV	60.6%	NA	792

Sharif (2009)	Pakistan	Cross-sectional	100	NA	56.0%	HBV 29%	NA	NA	EV	50.0%	NA	2200
						HCV 71%						

Baig (2008)	India	Prospective	150	51	84.0%	Alcohol 48.7% HBV 26% HCV 9.3% Others 7.3% Unknown 8.7%	NA	A, 64.7% B, 21.3% C, 14%	EV	70.7%	46.7%	1014 (optimal) 909

Parrino (2008)	Italy	NA	158	66.6 ± 9.6	63.3%	NA	0%	A, 64%	EV	74.1%	19.6%	1300
								B, 31%				
								C, 5%				

Tao (2008)	China	NA	69	53	63.8%	HBV 100%	NA	A, 20.3%	EV	78.3%	NA	909
								B, 63.8%				
								C, 15.9%				

Yu (2008)	China	Retrospective	89	53	82.0%	HBV 100%	NA	A, 29.2%	EV	75.3%	29.2%	909
								B, 42.7%				
								C, 28.1%				

Lei (2007)	China	Retrospective	326	55	77.3%	Alcohol 4.3% HBV 90.8% HBV and HBV 1.5% Others 3.4%	NA	A, 14.7% B, 67.5% C, 17.8%	EV	41.7%	15.6%	0.55 (optimal) 0.42

Giannini (2006)	Italy	Prospective	218	58/54^*∗*^	58.7%	Alcohol 18.8%	8.7%	A, 50.9% B, 34.4% C, 14.7%	EV	54.1%	21.6%	909
						Viral hepatitis 48.2%						
						PBC 16.1%						
						Viral hepatitis and alcohol 12.4%						
						Others 4.6%						

Legasto (2006)	Philippines	Cross-sectional	150	51/57^*∗*^	70.7%	Alcohol 90% HBV 10%	0%	NA	EV	46.0%	NA	160 (optimal) 909

Sethar (2006)	Pakistan	Cross-sectional	113	37.1 ± 14.85	69.0%	HBV 33.6%	NA	A, 13.3% B, 60.2% C, 26.6%	EV	58.4%	NA	1445
						HCV 52.2%						
						HBV + HCV 7.1%						
						Others 7.1%						

Giannini (2005)	Italy	Prospective	68	65 ± 10	63.2%	Viral, 77.9%	NA	A, 35.3%	EV	40.0%	5.9%	909
						Nonviral, 22.1%		B, 41.2%				
								C, 23.5%				

Pleština (2005)	Croatia	NA	99	53.6 ± 9.76	78.8%	HCV/HBV 13.1% Alcohol 83.9% PBC 3%	0%	A, 20.2%	EV	100.0%	53.5%	NA
								B, 53.5%				
								C, 25.3%				
								Unknown, 1%				

Giannini (2003)	Italy	Retrospective	145	61	71.0%	Alcohol 16.6% HBV 11% HCV 53.8% Others 18.6%	NA	A, 37% B, 36% C, 27%	EV	61.0%	20.0%	909
			121	64	65.3%	Alcohol 24%	NA	A, 41.3% B, 42.1% C, 16.5%	EV	58.7%	15.7%	909
						HBV 5%						
						HCV 63.6%						
						Others 7.4%						

EV, esophageal varices; GV, gastric varices; HBV, hepatitis B virus; HCC, hepatocellular carcinoma; HCV, hepatitis C virus; NA, not available; PBC, primary biliary cirrhosis; Pts, patients.

*Notes*. *∗*, age of patients was applied according to grade of varices or severity of cirrhosis.

**Table 2:** Results of meta-analyses in subgroups for any varices.

Groups	AUSROC	Sensitivity (95% CI)	Specificity (95% CI)	PLR (95% CI)	NLR (95% CI)	DOR (95% CI)
Threshold of 909	0.8867	0.84 (0.82–0.86)	0.80 (0.78–0.82)	3.95 (2.66–5.86)	0.21 (0.13–0.32)	25.06 (11.84–53.03)
Patients with viral hepatitis	0.8675	0.92 (0.90–0.94)	0.78 (0.74–0.81)	3.80 (2.04–7.08)	0.11 (0.06–0.23)	37.76 (14.43–98.84)
High quality studies	0.876	0.84 (0.81–0.87)	0.77 (0.74–0.8)	3.64 (2.11–6.3)	0.15 (0.07–0.33)	23.79 (10.35–54.7)
Prospective studies	0.8748	0.86 (0.84–0.88)	0.76 (0.73–0.79)	3.59 (2.39–5.39)	0.12 (0.07–0.22)	33.85 (15.67–73.15)
Region						
Europe	0.8289	0.83 (0.8–0.86)	0.65 (0.61–0.7)	2.58 (1.81–3.69)	0.2 (0.11–0.38)	15.46 (7.07–33.79)
Asia	0.9195	0.86 (0.84–0.87)	0.86 (0.84–0.88)	5.18 (3.5–7.65)	0.11 (0.06–0.2)	55.48 (24.27–126.81)
Africa	0.8537	0.87 (0.84–0.9)	0.71 (0.64–0.77)	3.28 (1.74–6.16)	0.16 (0.08–0.34)	23.31 (9.2–59.09)
North America	NA	0.82 (0.75–0.88)	0.67 (0.56–0.77)	2.45 (1.76–3.4)	0.27 (0.18–0.39)	9.06 (4.73–17.36)
Sample size						
<100	0.7895	0.81 (0.77–0.84)	0.7 (0.65–0.75)	2.57 (2.01–3.3)	0.25 (0.15–0.4)	12.58 (6.34–24.97)
≥100	0.9012	0.85 (0.83–0.86)	0.79 (0.77–0.81)	4.1 (2.92–5.77)	0.14 (0.09–0.22)	34.51 (18.84–63.2)
Prevalence of varices						
<50%	0.8804	0.91 (0.88–0.93)	0.86 (0.83–0.88)	5.29 (3.03–9.23)	0.11 (0.04–0.3)	54.16 (14.29–205.25)
≥50%	0.8633	0.83 (0.81–0.84)	0.73 (0.7–0.75)	3.15 (2.45–4.05)	0.19 (0.13–0.26)	21.12 (12.85–34.71)

AUSROC, area under the summary receiver operating characteristic curves; CI, confidence interval; DOR, diagnostic odds ratios; NLR, negative likelihood ratio; PLR, positive likelihood ratio.

## Abbreviations

PSR: Platelet count to spleen diameter ratio
HCC: Hepatocellular carcinoma
TP: True positive
FP: False positive
FN: False negative
TN: True negative
AUSROC: Area under the summary receiver operating characteristic curve
SE: Standard error
CI: Confidence interval
PLR: Positive likelihood ratio
NLR: Negative likelihood ratio
DOR: Diagnostic odds ratio.
