# Supplementary material for: Platelet Count to Spleen Diameter Ratio for the Diagnosis of Gastroesophageal Varices in Liver Cirrhosis: A Systematic Review and Meta-Analysis
Source: Gastroenterol Res Pract. 2017 Feb 8;2017:7407506. doi: 10.1155/2017/7407506 (PMC5320338; doi:10.1155/2017/7407506)
Supplement: Supplementary file 1 — Supplementary Material includes two parts. Supplementary Table 1 described the diagnostic value of included studies. Supplementary Table 2 described the QUADAS-2 results. [file 7407506.f1.zip › Supplementary Table 1_GRP_1819155.docx]

| **Supplementary Table 1. The diagnostic value of included studies** | | | | | | | | | |
| --- | --- | --- | --- | --- | --- | --- | --- | --- | --- |
| **First author (Year)** | **Cut-off of varices** | **Any size varices** | | | | **High-risk varices** | | | |
|  |  | **TP** | **FP** | **FN** | **TN** | **TP** | **FP** | **FN** | **TN** |
| Karatzas A (2016) | 1310.597 (optimal) | 19 | 9 | 5 | 5 | NA | NA | NA | NA |
|  | 909 | 13 | 9 | 11 | 5 | NA | NA | NA | NA |
| Kim TY (2016) | 860 | 25 | 11 | 15 | 52 | 11 | 18 | 2 | 72 |
| Takuma Y (2016) | 3.36 | 23 | 8 | 1 | 28 | 15 | 7 | 1 | 37 |
| Xu XD (2016) | 1004 (optimal) | 81 | 24 | 14 | 117 | NA | NA | NA | NA |
|  | 909 | 71 | 18 | 24 | 123 | NA | NA | NA | NA |
| Albreedy AM (2015) | 979.9 | 66 | 24 | 0 | 10 | 47 | 9 | 0 | 10 |
| Cho EJ (2015) | NA | NA | NA | NA | NA | 51 | 75 | 22 | 71 |
| Stefanescu H (2015) | NA | NA | NA | NA | NA | 32 | 15 | 15 | 28 |
| Valero G (2015) | 1.86 | 74 | 9 | 12 | 6 | NA | NA | NA | NA |
| Zhao D (2015) | 909 | 47 | 13 | 17 | 47 | NA | NA | NA | NA |
| Chiodi D (2014) | 1010 | 59 | 13 | 20 | 33 | 33 | 27 | 20 | 45 |
| Gonzalez-Ojeda A (2014) | 884 | 61 | 5 | 12 | 13 | NA | NA | NA | NA |
| Wang LJ (2014) | NA | NA | NA | NA | NA | 45 | 11 | 9 | 39 |
| Zafar S (2014) | 909 | 124 | 4 | 7 | 80 | NA | NA | NA | NA |
| Calvaruso V (2013) | 800 | 40 | 13 | 14 | 29 | 19 | 25 | 7 | 45 |
| Masjedizadeh AR (2013) | 663 | 56 | 5 | 63 | 16 | NA | NA | NA | NA |
| Rajendran PA (2013) | NA | NA | NA | NA | NA | 56 | 1 | 10 | 34 |
| Saad Y (2013) | 545 | 17 | 2 | 3 | 10 | 9 | 4 | 1 | 18 |
| Sharma P (2013) | 1023.2 (optimal) | 94 | 15 | 30 | 35 | NA | NA | NA | NA |
|  | 909 | 79 | 12 | 45 | 38 | NA | NA | NA | NA |
| Al-Dahshan M (2012) | 1023 | 32 | 6 | 8 | 14 | NA | NA | NA | NA |
| Colecchia A (2012) | 1883 (optimal) | 52 | 35 | 1 | 12 | NA | NA | NA | NA |
|  | 513 | 14 | 1 | 39 | 46 | NA | NA | NA | NA |
| Mahassadi AK (2012) | 868 (training sample) | 70 | 6 | 15 | 20 | 66 | 12 | 12 | 21 |
|  | 868 (validation sample) | 40 | 4 | 32 | 15 | 38 | 8 | 22 | 23 |
| Mangone M (2012) | 936.364 (optimal) | 20 | 20 | 11 | 36 | NA | NA | NA | NA |
|  | 909 | 18 | 19 | 13 | 37 | NA | NA | NA | NA |
| Nisar S (2012) | 909 | 98 | 3 | 4 | 45 | NA | NA | NA | NA |
| Abu El Makarem MA (2011) | 939.7 | 131 | 6 | 0 | 38 | NA | NA | NA | NA |
| Agha A (2011) | 885 | 31 | 1 | 0 | 11 | NA | NA | NA | NA |
| Cherian JV (2011) | 666 | 118 | 10 | 60 | 41 | NA | NA | NA | NA |
| Esmat S (2011) | 1326.6 (optimal) | 79 | 3 | 3 | 15 | 56 | 24 | 4 | 16 |
|  | 909 | 69 | 3 | 13 | 15 | NA | NA | NA | NA |
| Mosqueira JR (2011) | 909 | 14 | 3 | 21 | 9 | 11 | 6 | 11 | 19 |
| Stefanescu H (2011) | 1068 | 90 | 6 | 26 | 15 | NA | NA | NA | NA |
| Barikbin R (2010) | 921 | 36 | 4 | 1 | 9 | NA | NA | NA | NA |
| Mattos AZ (2010) | 909 | 93 | 24 | 27 | 20 | NA | NA | NA | NA |
| Nashaat EH (2010) | 820 | 37 | 3 | 7 | 3 | NA | NA | NA | NA |
| Sarangapani A (2010) | NA | NA | NA | NA | NA | 45 | 9 | 6 | 46 |
| Schwarzenberger E (2010) | 909 | 61 | 21 | 15 | 40 | NA | NA | NA | NA |
| Agha A (2009) | 909 | 154 | 5 | 0 | 152 | NA | NA | NA | NA |
| Barrera F (2009) | NA | NA | NA | NA | NA | 25 | 9 | 8 | 25 |
| Camma C (2009) | 792 | 52 | 16 | 11 | 25 | NA | NA | NA | NA |
| Shairf MA (2009) | 2200 | 49 | 6 | 1 | 44 | NA | NA | NA | NA |
| Baig WW (2008) | 1014 (optimal) | 104 | 5 | 2 | 39 | NA | NA | NA | NA |
|  | 909 | 85 | 5 | 21 | 39 | NA | NA | NA | NA |
| Parrino A (2008) | 1300 | 60 | 3 | 57 | 38 | NA | NA | NA | NA |
| Tao W (2008) | 909 | 54 | 7 | 0 | 8 | NA | NA | NA | NA |
| Yu JY (2008) | 909 | 60 | 4 | 7 | 18 | NA | NA | NA | NA |
| Lei JB (2007) | 0.55 (optimal) | 132 | 15 | 4 | 175 | NA | NA | NA | NA |
|  | 0.42 | 122 | 8 | 14 | 182 | NA | NA | NA | NA |
| Giannini EG (2006) | 909 | 108 | 33 | 10 | 67 | NA | NA | NA | NA |
| Legasto GM (2006) | 160 (optimal) | 61 | 16 | 8 | 65 | NA | NA | NA | NA |
|  | 909 | 42 | 4 | 27 | 77 | NA | NA | NA | NA |
| Sethar GH (2006) | 1445 | 66 | 0 | 0 | 47 | NA | NA | NA | NA |
| Giannini EG (2005) | 909 | 27 | 11 | 0 | 30 | NA | NA | NA | NA |
| Plestina S (2005) | NA | NA | NA | NA | NA | 37 | 19 | 16 | 27 |
| Giannini E (2003) | 909 (training sample) | 89 | 4 | 0 | 52 | NA | NA | NA | NA |
|  | 909 (validation sample) | 71 | 29 | 0 | 21 | NA | NA | NA | NA |
| ***Abbreviations:*** NA, not available; TP, true positive; FP, false positive; FN, false negative; TN, true negative | | | | | | | | | |
