# Supplementary material for: Platelet Count to Spleen Diameter Ratio for the Diagnosis of Gastroesophageal Varices in Liver Cirrhosis: A Systematic Review and Meta-Analysis
Source: Gastroenterol Res Pract. 2017 Feb 8;2017:7407506. doi: 10.1155/2017/7407506 (PMC5320338; doi:10.1155/2017/7407506)
Supplement: Supplementary file 1 — Supplementary Material includes two parts. Supplementary Table 1 described the diagnostic value of included studies. Supplementary Table 2 described the QUADAS-2 results. [file 7407506.f1.zip › Supplementary Table 2_GRP_1819156.docx]

| **Supplementary Table 2. QUADAS-2 Results** | | | | | | | | |
| --- | --- | --- | --- | --- | --- | --- | --- | --- |
| **First author, Journal (Year)** | **Risk of Bias** | | | |  | **Applicability Concerns** | | |
|  | **Patient Selection** | **Index Test** | **Reference Standard** | **Flow and Timing** |  | **Patient Selection** | **Index Test** | **Reference Standard** |
| Karatzas A (2016) | UR | UR | UR | LR |  | HC | HC | HC |
| Kim TY (2016) | LR | LR | UR | LR |  | HC | HC | HC |
| Takuma Y (2016) | UR | LR | LR | LR |  | HC | HC | HC |
| Xu XD (2016) | LR | LR | LR | LR |  | LC | HC | HC |
| Albreedy AM (2015) | UR | UR | LR | LR |  | UC | HC | HC |
| Cho EJ (2015) | UR | UR | UR | UR |  | HC | HC | HC |
| Stefanescu H (2015) | LR | UR | UR | UR |  | HC | HC | HC |
| Valero G (2015) (abstract) | UR | UR | UR | UR |  | HC | HC | HC |
| Zhao D (2015) | UR | UR | UR | UR |  | HC | HC | HC |
| Chiodi D (2014) | UR | UR | UR | UR |  | HC | HC | HC |
| Gonzalez-Ojeda A (2014) | UR | UR | UR | UR |  | HC | HC | HC |
| Wang LJ (2014) | UR | UR | UR | UR |  | HC | HC | HC |
| Zafar S (2014) | UR | UR | UR | UR |  | UC | HC | HC |
| Calvaruso V (2013) | LR | UR | UR | LR |  | HC | HC | HC |
| Masjedizadeh AR (2013) | UR | UR | UR | UR |  | HC | HC | HC |
| Rajendran PA (2013) (abstract) | LR | UR | UR | UR |  | HC | HC | HC |
| Saad Y (2013) | UR | UR | UR | UR |  | HC | HC | HC |
| Sharma P (2013) | LR | UR | UR | LR |  | HC | HC | HC |
| Al-Dahshan M (2012) | UR | UR | UR | UR |  | HC | HC | HC |
| Colecchia A (2012) | LR | LR | LR | LR |  | HC | HC | HC |
| Mahassadi AK (2012) | LR | UR | UR | UR |  | HC | HC | HC |
| Mangone M (2012) | LR | LR | LR | UR |  | HC | HC | HC |
| Nisar S (2012) | UR | UR | UR | UR |  | UC | HC | HC |
| Abu El Makarem MA (2011) | LR | UR | LR | UR |  | HC | HC | HC |
| Agha A (2011) | LR | UR | UR | UR |  | LC | HC | HC |
| Cherian JV (2011) | LR | UR | LR | UR |  | HC | HC | HC |
| Esmat S (2011) | UR | UR | UR | UR |  | HC | HC | HC |
| Mosqueira JR (2011) | UR | UR | UR | LR |  | HC | HC | HC |
| Stefanescu H (2011) | UR | UR | UR | LR |  | HC | HC | HC |
| Barikbin R (2010) | LR | LR | LR | LR |  | HC | HC | HC |
| Mattos AZ (2010) | UR | UR | UR | HR |  | HC | HC | HC |
| Nashaat EH (2010) | UR | UR | UR | UR |  | HC | HC | HC |
| Sarangapani A (2010) | UR | UR | UR | LR |  | HC | HC | HC |
| Schwarzenberger E (2010) | UR | UR | UR | UR |  | HC | HC | HC |
| Agha A (2009) | LR | LR | LR | UR |  | HC | HC | HC |
| Barrera F (2009) | UR | UR | UR | UR |  | HC | HC | HC |
| Camma C (2009) | LR | UR | LR | LR |  | HC | HC | HC |
| Shairf MA (2009) | UR | UR | UR | UR |  | HC | HC | HC |
| Baig WW (2008) | UR | UR | LR | UR |  | HC | HC | HC |
| Parrino A (2008) | LR | LR | UR | LR |  | HC | HC | HC |
| Tao W (2008) | UR | UR | UR | UR |  | HC | HC | HC |
| Yu JY (2008) | UR | UR | UR | LR |  | HC | HC | HC |
| Lei JB (2007) | UR | UR | UR | LR |  | HC | HC | HC |
| Giannini EG (2006) | LR | LR | LR | HR |  | HC | HC | HC |
| Legasto GM (2006) | UR | UR | LR | UR |  | HC | HC | HC |
| Sethar GH (2006) | UR | UR | UR | UR |  | HC | HC | HC |
| Giannini EG (2005) | UR | LR | LR | LR |  | HC | HC | HC |
| Plestina S (2005) | UR | UR | UR | UR |  | HC | HC | HC |
| Giannini E (2003) | UR | UR | UR | UR |  | HC | HC | HC |
| Abbreviations: LR, low risk; HR: high risk, UR; unclear risk; LC, low concern; HC, high concern; UC, unclear concern. | | | | | | | | |
